# How personality affects reaction. A mental health behavioral insight review during the Pandemic

**DOI:** 10.1007/s12144-021-02425-9

**Published:** 2021-11-03

**Authors:** Evangelos Mourelatos

**Affiliations:** 1 Department of Economics, Oulu Business School, University of Oulu, Oulu, Finland; 2 Department of Economics, University of Patras, Patras, Greece

**Keywords:** Covid-19, Pandemic, Individual differences, Big Five Personality traits, Anxiety, Depression, Behavior

## Abstract

The fear caused by the Covid-19 pandemic is changing our psychology and behavior. This ongoing negative event, imposing restrictions such as home isolation and social distancing, can result in heightened anxiety, depression and a sense of loneliness, with immediate effects on mental health. This study investigates adolescents’ reaction to the pandemic, by analyzing the behavioral mental health trends of depression, anxiety and sense of loneliness, in relation to personality traits. After controlling for demographics and family background, our results reveal strong relationships between several personality traits and psychological health indicators, during the pandemic in Greece. A total of 419 secondary school students (aged 12–18) were administered the State-Trait Anxiety Inventory for Children (STAIC), the Child Depression Inventory (CDI), the Big Five Inventory for measuring personality (BFI) and the Children’s Loneliness Questionnaire (CLS) during two time periods within pandemic. Overall, it appears that depression increased significantly in line with the escalation of the pandemic, while anxiety decreased, with the strongest predictors being the personality variables of extraversion, neuroticism and openness. Surprisingly, the study also revealed that the level of extraversion has a positive effect on changes in anxiety, while a negative one on changes in depression. On the other hand, neuroticism and openness seem to negatively correlate with anxiety changes and positively with depression changes. These findings highlight the importance of considering these variables in addressing individuals’ mental health behavior during the Covid-19 pandemic and elucidate the literature by offering a deeper understanding of the strong relationship between personality, depression and anxiety.

## Introduction

As the number of cases increased and the consequences of the global spread of coronavirus disease (Covid-19) began to be felt, governments all over the world reacted by adopting isolation measures. During the Pandemic, an estimated 2.6 billion people – one-third of the world’s population – was living under some kind of lockdown or quarantine, with many believing that this is arguably the largest psychological experiment ever conducted. Thus, home isolation and social distancing emerged as the most effective ways for societies to restrict the Covid-19 pandemic. In short, and perhaps unsurprisingly, people who are quarantined are very likely to develop a wide range of symptoms of psychological stress, including low mood, insomnia, stress, anxiety, anger, irritability, emotional exhaustion, depression and post-traumatic stress symptoms (Cruwys et al., 2020; Nikelly, 2001). Already, in China, this stress-related situation and its expected mental health effects are being reported in the first research papers about the lockdown (Duan & Zhu, 2020; Qiu et al., 2020 and Bo et al., 2020). Stressful negative life events, such as the Covid-19 pandemic, are known to contribute to the development of anxiety, depression and loneliness levels, especially in vulnerable social groups such as students and the elderly (Williamson et al., 1995; Joiner et al., 1999, Garnefski et al., 2001 and Xiong et al., 2020). Initial studies, using a large web survey on approximately 8,000 participants, revealed that poor housing is associated with an increased risk of depressive symptoms during lockdown and poor working performance from home were over four times more likely to also report depression (Amerio et al., 2020).

All the aforementioned psychological morbidities obviously have a link with an individual’s personality structure. For example, Eysenck’s personality theory highlights the relationship between Neuroticism, Extraversion and Psychoticism with different kinds of behaviors during a stressful negative event (Eysenck, 1947, 1952;1985; Deary et al., 1998). Moreover, many cognitive vulnerability theories also point out the essential role of personality characteristics in gaining a deep understanding of the mechanisms and processes causing loneliness, anxiety and depression, especially in youth (Abela & Hankin, 2008). However, the effects of personality traits on the psychological response to negative life events and their influence on individuals’ psychological health is currently under investigation (Goodman et al., 2017; Kotov et al., 2010).

To the best of our knowledge, our article belongs to the first attempts to investigate the behavioral aspects of adolescents that emerge through the Covid-19 pandemic. This study examines the relationship between the Big Five personality factors and several mental health conditions such as the sense of loneliness, anxiety levels, and depression in adolescents, specialized in the mood factors of adolescents (Howarth & Schokman-Gates, 1989). In contrast to previous studies, we elaborated several data in panel form (i.e. two time periods), and, using the established trait theory of personality, we make a first attempt to explain how girls and boys start having feelings of loneliness, anxiety and depression as a consequence of changes in their human behavior (i.e. home isolation and social distancing), as their best defense in tackling the virus, due to their personality characteristics. By utilizing ordinary least squares (i.e. OLS) regression analysis, we integrate and expand on central findings from basic research in loneliness, anxiety, depression and personality to articulate an elaborated scientific report that can explain adolescents’ mental health behavioral insights related to the Covid-19 pandemic. Thus, the contribution of the present study is twofold. First, we explored the incremental value of Extraversion, Agreeableness, Conscientiousness, Neuroticism, and Openness to Experience in predicting students’ loneliness, anxiety and depression levels, controlling for demographics, family background and social economic characteristics. Secondly, by incorporating interaction terms in our analysis, we explore the exact student profile (i.e. based on personality, demographics and cognitive attributes) that was most affected by the Covid-19 pandemic, in terms of anxiety and depression.

Our analysis revealed that Extraversion, Neuroticism and Openness are crucial for both depression- and anxiety-related behavioral changes due to Covid-19. More specifically, extraversion has a robust significantly positive relationship with the percentage change of anxiety and a negative effect with the percentage change of depression, while neuroticism and openness follow the opposite direction. Our results provide initial evidence that several personality indicators can explain and predict adolescents’ mental health behavior related to major negative events, such as the Covid-19 pandemic (Ayub, 2015). This contribution relies on the economic considerations group of studies, for social distancing and behavioral based policies during an epidemic by analyzing the role of the non-cognitive traits on health behaviors in adolescence (Fenichel, 2013). In this context, a growing partnership between economists and psychologists is crucial in mapping incentives through micro-level behaviors to macro-level outcomes for health and social welfare measurements. Each field has a lot to learn from the other, with recent evidence showing that the sources of individual differences in our behavioral profile need a new way of thinking. Thus, as economists developed a deeper understanding of the determinants of personality, they discovered that multiple traits play an important role in an individual’s behavioral profile, are required in various life tasks and play an important role in individuals’ reactions to exogenous life events (Heckman et al., 2019).

## Related Literature and Hypotheses

Let’s start with the obvious. Any infectious disease epidemic, such as the new coronavirus strain, is scary; understandably, people will be frightened and will try a behavioral adaptation to these new living circumstances. This behavioral procedure has been a part of human response to infectious diseases for millennia. The World Health Organization (2006), governments (Stern & Markel, 2009), and public health experts (Ferguson et al., 2005; Glass et al., 2006) have emphasized the potential importance of public policies designed to elicit behavioral changes in preparing for and responding to infectious disease epidemics. Specifically, these strategies provide motivations, some quite strong, to reduce interpersonal contacts through social distancing and home isolation policies (Halloran et al., 2008). Hence, the occurrence of this negative life event (i.e. the Covid-19 pandemic), raised similar questions, regarding the determinants that may explain the variation in several population groups’ behavioral insights (Hoppe et al., 2018; Paz & Amir, 1974). Under these new circumstances, psychologists such as Metin Başoğlu, a professor of psychiatry and founder of the Istanbul Center for Behavior Research & Therapy, has studied the emotional and behavioral response of earthquake survivors and sees parallels in today’s reactions to the coronavirus (Basoglu and Salcioglu, 2011). However, in general, few studies have focused on investigating the role of personality during the first stages of pandemic-induced confinement. López-Núñez et al., 2021, surveying Spanish adults regarding the relevance of social/work status, found several correlations between conscientiousness, extraversion and emotional stability (i.e. the opposite of neuroticism) with anxiety, depression and life satisfaction. In the same direction, Lippold et al., 2020, launching an online survey in several European countries during the first stages of the Covid-19 pandemic, and Qian & Yahara, 2020 with a sample from Japan, revealed a robust relationship between neuroticism and the level of perceived threat by the coronavirus. Nikčević et al., 2021, analyzing a sample of United States residents, show that several traits appear to protect against Covid-19 psychological distress. Some studies have also explored the link between individuals’ personality characteristics, social distancing, hygiene (Abdelrahman, 2020) and adherence to life restrictions due to the virus (Zajenkowski et al., 2020).

In our study, we tried to interpret the behavioral response of adolescents dynamically, with two waves of surveying the same participants, in terms of their level of loneliness, anxiety and depression during the Covid-19 pandemic (Laliotis, 2020). By adopting the Big Five personality theory and utilizing a simplified cognitive vulnerability-stress model, we investigate how this negative event contributes to several psychological disorders and symptoms and how it varies due to differences in personality (Proto & Zhang, 2021). All personality theories have at least one dimension representing the predisposition of sensitivity to a negative stimulus, and thus a vulnerability for anxiety disorders (Caspi et al., 2005). Finally, we know that genetic and environmental factors account for about 50% of the variance in personality (Plomin & Asbury, 2005). Hence, from a theoretical perspective, personality factors are a most promising starting point for addressing questions about individual differences in response and behavior, because personality is defined as the predisposition to respond to a certain class of stimuli with a certain class of behaviors, and these stimulus–response configurations are stable over time (Montag & Reuter, 2014). Therefore, it can be assumed that people with high scores on several personality levels related to depression or anxiety are more prone to react with panic to the coronavirus. For adolescents in particular, the existence of depression and anxiety is, in general, associated with poor wealth and behavioral outcomes, including a higher risk of disruptive behaviors, sensitivity and greater likelihood of being involved in unpleasant situations (Fletcher, 2008; Saluja et al., 2004). Many studies also revealed that depression and anxiety during adolescence may also be linked to occurrence of negative life events, which may lead to decreased human capital accumulation, which would have negative implications for lifetime income, occupational options, and socioeconomic status (Ettner et al., 1997; Hamilton et al., 1997; Kessler et al., 1995). Qualitative and quantitative reviews have also suggested that the prevalence of loneliness, depressive and anxiety symptoms vary due to for the emergence of gender differences (Bebbington, 1996). For example, Hankin et al., 1998, 2007; Hankin and Abramson, 2001, 2001; Nolen-Hoeksema & Girgus, 1994, revealed that these disorders increase from childhood into adolescence but only among girls and not among boys. Another community cross-sectional study found that more girls than boys displayed higher levels of a mixed anxiety-depressive syndrome, and this effect was especially pronounced among referred adolescents (Compas et al., 1997). Research also shows (Nolen-Hoeksema, 1990; Petersen et al., 1991; Nolen et al., 1994, 1999) that more girls than boys have anxiety disorders, and this often precedes the onset of a depressive disorder and has been linked to greater peer rejection, loneliness, and depression (Crick & Grotpeter, 1995). Thus, existing evidence indicates that more girls than boys develop loneliness, depressive and anxiety symptoms and disorders, and this gender difference emerges and increases during a major negative life event, such as the Covid-19 pandemic in our study and the life changes entailed (i.e. home isolation and social distancing).

Taking into consideration the above-mentioned studies during the pandemic and the foregoing general personality papers and investigations,^1^ our main research question focuses on personality traits and their effects on health issues such as anxiety and depression, during the Covid-19 pandemic, for adolescent participants. We develop the following hypotheses based on the above studies and the nature of the personality characteristics examined:

- *H1. There will be differences between males and females concerning the percentile change in anxiety and depression due to the Pandemic.*
- *H2. A high level of neuroticism will be associated with a high percentile change in depression, especially for females.*
- *H3. Extraversion will be negatively associated with depression and positively with anxiety levels according to the specificity of a pandemic.*
- *H4. Conscientiousness, agreeableness and openness may have slightly debatable effects on anxiety and depression, but they are not the key factors in behavioral change.*
- *H5. Grades will be a moderator for the effects of the above-mentioned personality effects on anxiety and depression during the pandemic.*

## Methodology and Procedures

When the Greek government announced the closure of all public schools from 10 March to 11 May 2020, following the confirmation of the first three cases of Covid-19 in Greece on 27 February, we sent an online questionnaire randomly to secondary school students through the online systems of private supplementary tutoring schools.^2^ The study consisted of two consecutive phases held over a month. Our data were collected, after the distribution of the questionnaire to potential participant students, during a two-time period by having the pre and the post period of the Pandemic restrictions (i.e. from the government), in order to efficiently investigate online behavioral changes.

Our questionnaire was distributed randomly to students through emails with an embedded link to our survey in the early period of the pandemic in Greece. In the beginning of the questionnaire, participants reported the exact date and time they started this and their ID number, to provide us with unique identifiers (as participation was anonymous). After that, they reported their demographics (gender, age, region of residence) and some cognitive skills such as average grades in the current school year and average time of internet usage. Students then outlined their family structure, through questions including number of siblings, type of family (i.e. nuclear, single parent or grandparent-other relative form) and environment characteristics (rural, suburban or urban area of residence). Next, we measured the students’ social economic background (SES). Based on the available literature on the effects of SES on children’s emotional status (Akee et al., 2018) we needed a group of questions to get an indication of each participant’s SES level. We thus used the widely recognized Family Affluence Scale (FAS), which is used in many studies measuring wealth (Boyce et al., 2006) that has been characterized as a valid measure of socio-economic status levels, easy for adolescents to answer (Currie et al., 1997). Furthermore, taking into consideration that many previous studies have linked an individual’s levels of life satisfaction to personality traits (Arrindell & Luteijn, 2000; Hong & Giannakopoulos, 1994), and have highlighted their relationship with depression and anxiety (Headey et al., 1993; Guney et al., 2010), we also embodied the Satisfaction with life scale (SWLS inventory) in our questionnaire (Heaven, 1989).

Next, we chose a middle time period between the closure and opening dates of Greek public schools, in order to investigate the effect of the pandemic on students’ levels of loneliness, depression and anxiety when at its peak (Garnefski et al., 2001). Twenty days after the start of the Covid-19-related restrictions, we started sending emails with the second wave of the survey to the initial participants, asking them to report only their average internet usage during the Covid-19 restrictions (i.e. we used Internet usage as a proxy index of students’ mobile and computer competence), the Anxiety, Depression Inventories and we embedded the Loneliness inventory, in order to have one more outcome reflecting the participants’ mental health profile in relation to the Pandemic. In the first wave we used the T-Anxiety and in the second wave the S-Anxiety inventory respectively. Regarding depression and personality traits, the same set of questions was used.

Our final number of participants was 419 secondary education students, as 116 of the initial participants did not respond to our second wave of questions. As our target group was students aged 12–18 years, we choose an online self-reported questionnaire because adolescents can report accurately on their own depressed mood and symptoms (Kazdin, 1994) and can readily recognize various different emotions (positive/negative valence and self/other perspectives) after age 9 (Harter, 1999). Adolescents might be the best informants about their own affect after age 9, when they can recognize and identify different emotions.

**Fig. 1:**
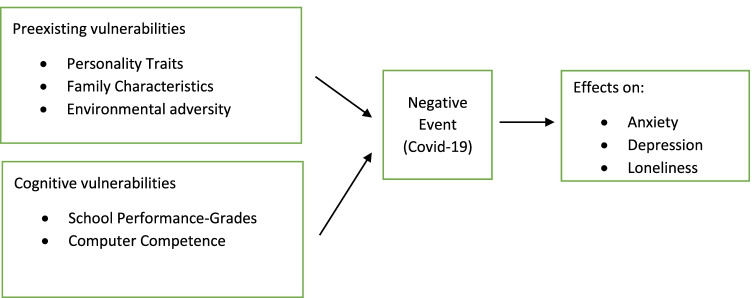
Schematic diagram of the proposed, general elaborated cognitive vulnerability-transactional stress model of anxiety, depression and loneliness

Thus, to provide a framework for our analysis, we describe an interdisciplinary and simplified approach of the cognitive vulnerability–stress model under a global and major negative event. The model is linked to students’ personality traits, family characteristics, environmental adversity and cognitive skills and generates predictions about depression, anxiety and loneliness levels during a negative—stressful incident (Hankin, 2008). We also use the framework to discuss potential gender differences (Fig. 1).

## Material and Method

### Participants

Our sample consisted of 241 (57.5%) female students, with an average age of 16 years. Based on age, we grouped the sample into Junior High School (aged 12–15 years) and General High School (aged 16–18 years) students. We had 311 students from General High School (74.22%). Half of these (49.4%) came from urban areas. 351 students came from nuclear families (with both parents), 60 from single-parent families (83.77% and 14.32% respectively) and only 8 students reported that they were living with other relatives (1.91%). Lastly, we had 45 single child students (10.74%), while 374 students (54.18%) reported at least one brother or sister.

A detailed overview of the sample characteristics can be found in Table 1, which presents the descriptive statistics for the explanatory variables grouped in four groups; demographic characteristics, cognitive skills, socioeconomic background (including family characteristics) and the students’ personality traits – soft skills.

**Table 1:** Descriptive Statistics

Variable	Mean	SD	Min	Max	N
*Demographic Characteristics*					
Female (0/1)	0.575	0.495	0	1	419
Age	16.38	1.475	12	18	419
High School (%)					
Junior	25.78				108
General	74.22				311
*Cognitive Characteristics*					
Grades	17.57	1.810	7.6	20	419
Internet Increase – Computer/Smartphones Competence	3.61	2.598	-5	17	419
*Personality Traits (Big Five)*					
Openness	3.552	0.546	1.7	4.9	419
Conscientiousness	3.351	0.594	1.556	4.889	419
Extraversion	3.173	0.606	1.25	4.75	419
Agreeableness	3.640	0.494	1.556	5	419
Neuroticism	2.896	0.658	1.125	5	419
*Social Economic Background*					
Satisfaction with life index (SWLS)	21.568	6.284	5	34	419
Family Affluence index (FAS)	12.089	1.831	5	18	419
Urban Family Area (0/1)	0.494	0.501	0	1	419
Parental Status (%)					
Both Parents	83.77				351
Single Mother	11.69				49
Single Father	2.63				11
Other Relative	1.91				8
Number of Brothers and Sisters (%)					
0	10.74				45
1	54.18				227
2	24.58				103
3	7.16				30
4	2.15				9
5 and more	1.19				5

Dataset with results drawn from the online Questionnaire. Author’s calculation

Internet Increase is measured in hours. Big Five personality traits are on normalized values. The reference category for Urban family areas is the Rural family area

### Measures

Participants’ information, including personality, anxiety, depression and loneliness levels were also obtained.

#### Personality

In respect to personality traits, students were asked to fulfill the Big Five Personality test inventory. This test is a questionnaire with a 44-item inventory (John & Srivastava, 1999; McCrae & Costa, 1999), measuring each personality trait i.e., Openness, Conscientiousness, Extraversion, Agreeableness, Neuroticism (hereafter OCEAN). The role of personality traits is well established in standard models of individual behavior regarding individuals’ performance (Heckman et al., 2019) and the adopted OCEAN taxonomy captures individual-specific differences in thinking, feeling, and behaving (Filiz-Ozbay, et al., 2018). In addition, this mid-sized test ensures an accurate and stable measurement of each personality facet (Cobb-Clark & Schurer, 2012), without requiring an excessively long time, which could result in some measurement bias errors (John et al., 1991, 2008). The Big Five dimensions of personality were estimated on a scale of 1–5, where 1 = disagree, 2 = slightly disagree, 3 = neutral, 4 = slightly agree and 5 = agree (Goldberg, 1993). Initially, with respect to the personality profile, the five variables of the Big Five Personality test are designed to give a mean of 50, with a standard deviation of 10 for each trait (i.e. Openness, Conscientiousness, Extraversion, Agreeableness, Neuroticism). Afterwards, the OCEAN factors were constructed through factor analysis, in order for each trait to be orthogonal to the rest (McCrae & Costa, 1999). Hence, the normalized version of the OCEAN variables has a range of 1 to 5, where 1 denotes a very low and 5 a very high incidence of the trait.

Nevertheless, to allow for an easier interpretation of our estimates, Big Five scores in our analysis are standardized to have mean zero and standard deviation of one in all reported econometric specifications. Last but not least, internal consistency reliability was determined by calculating Cronbach’s alpha coefficient (i.e. Openness a = 0.72, Conscientiousness a = 0.73, Extraversion a = 0.68, Agreeableness a = 0.70, Neuroticism a = 0.70).

#### Depression

To estimate the effects on students’ current depression levels as a consequence of social distancing, quarantine and isolation due to Covid-19, we used the Children’s Depression Inventory (CDI), which is the most widely used and best studied scale for depressive symptoms in students (Kovacs & Preiss, 1992). Moreover, the CDI includes questions about the presence of specific symptoms over the past 2 weeks to screen students for depression (Helsel & Matson, 1984). Hence, the inventory has items pertaining to appearance, schoolwork, fatigue, eating habits, and aches and pains, all signs of depression which may be directly related to depressive feelings or thoughts during the previous 2 weeks due to a negative event (Hodges & Craighead, 1990). The CDI inventory consists of 27-item self-report rating instrument written at the lowest reading level of any measure of depression, designed specifically for adolescents. Each item has three response options, from which students select the one that most closely reflects their thoughts and feelings over the past two weeks. Each item receives a score of 0 to 2 points with a highest possible total score of 54. A score of 19 or above is in the clinical range for depression (Kovacs & Preiss, 1992). The CDI is also said to have five inner measured facets, which include negative mood, interpersonal problems, ineffectiveness, anhedonia, and negative self-esteem (Kovacs & Preiss, 1992). Additionally, similarly to our case, some studies used only 26 items of the CDI because school officials or review boards would not allow them to include item 9, concerning suicide ideation. For that reason, data points were recalculated to conform to the usual 27-item mean (the 26-item mean divided by 26, and the result added to the 26-item mean) (Twenge & Nolen –Hoeksema, 2002). In general, the CDI has demonstrated good internal consistency, reliability and moderate test–retest reliability (Saylor et al., 1984; Sitarenios & Stein, 2004). In our analysis, internal consistency reliability was determined by calculating Cronbach’s alpha coefficient (the CDI index had a Cronbach α = 0.85, with subscale reliabilities ranging from 0.846 to 0.871 for period T1 and a Cronbach α = 0.88, with subscale reliabilities ranging from 0.866 to 0.881 for period T2 respectively).

#### Anxiety

Regarding anxiety levels, we adopt the State-Trait Anxiety Inventory for Children (STAIC). The State-Trait Anxiety Inventory for Children (STAIC; Spielberger et al., 1973) is widely used to assess anxiety in young people (Beesdo et al., 2009). This instrument includes two independent scales: The State Scale, which aims to measure the current feelings of anxiety, and the Trait Scale, which assesses a more stable and long-lasting tendency to experience anxiety. State and trait anxiety are analogous in certain respects to kinetic and potential energy. S-Anxiety, like kinetic energy, refers to a palpable reaction or process taking place at a given time and level of intensity. T-Anxiety, like potential energy, refers to individual differences in reactions to major negative events. T-Anxiety may also reflect individual differences in the frequency and intensity with which a student experiences a threating situation (Hedl & Papay, 1982; Rodrigues et al., 2018). More particularly, the STAIC consists for a 40-item self-evaluation questionnaire which includes separate measures of state and trait anxiety (20 Likert scale items for each trait. This instrument used all original items with no modification whatsoever. The State-Anxiety scale (Current Anxiety index) consists of twenty statements that evaluate how respondents feel about anxiety “right now, at this moment” through four scales: one (not at all), two (somewhat), three (moderately so), and four (very much so). The Trait-anxiety scale consists of twenty statements that assess how people “generally feel” about anxiety with four scales: one (rarely), two (moderate), three (often). A rating of three indicates the presence of a high level of anxiety and one indicates the absence of a high level of anxiety (Spielberger et al., 1983). The anxiety level was found by calculating scores with a range from 20–60, the higher the score indicating greater anxiety (Spielberger et al., 1983). In a similar way, STAIC T-Anxiety scores are 3, 2, or 1 for all items. The T-Anxiety scale requires the subject to respond to each item by indicating the frequency of occurrence of the behavior described by it. The scoring weights are assigned to ‘very often’, ‘sometimes’, and ‘hardly ever’. Thus, the scores for each scale range from 20 (minimum) to 60 (maximum). Participants were asked to respond by ticking one of the three choices that described themselves best generally (usually) (T-Anxiety scale) (Dorr, 1981; Psychountaki et al., 2003). Taking into consideration the conceptual differences between languages (Liakos & Yannitsi, 1984), we adopted this Anxiety Inventory because, to the best of our knowledge, this inventory has been used in previous studies in Greece with high reliability and validity (Psychountaki et al., 2003). In our analysis internal consistency reliability was determined by calculating Cronbach’s alpha coefficient (the S-Anxiety index had an average Cronbach α = 0.91, with subscale reliabilities ranging from 0.903 to 0.908 and T-Anxiety index had an average Cronbach α = 0.90, with subscale reliabilities ranging from 0.895 to 0.903 concerning both periods).

Lastly, in our analysis we also constructed the Standardized Change of Anxiety (%) (PCA index) and Depression (PCD index), to investigate the determinants and the students’ specific characteristics that had a statistically significant effect on the behavioral change on anxiety and depression levels due to the pandemic. The mathematical formula of the standardized relative change was: (Current Anxiety (T2) – General Anxiety (T1))/ Standard Deviation of General Anxiety (T1)) and (Depression (T2) – Depression (T1))/ Standard Deviation of Depression (T1)).

#### Loneliness—Children’s Loneliness Questionnaire and Social Dissatisfaction Scale (CLQ)

The CLQ is a 24-item self-report instrument used to assess feelings of loneliness in children and adolescents (Asher et al., 1984). The questionnaire has 16 items that center around feelings of loneliness and social dissatisfaction, and eight items asking about hobbies and interests used as filler questions with 5-point Likert scale answers (Asher & Wheeler, 1985). The CLQ has been shown to be effective in identifying loneliness, particularly in identifying students’ reaction to major negative events (Asher & Wheeler, 1985; Cassidy & Asher, 1992). The scale has demonstrated reliability for use in students of varying ages from kindergarten through secondary school (Cassidy & Asher, 1992). Lastly, internal consistency reliability was determined by calculating Cronbach’s alpha coefficient (i.e. loneliness index had a Cronbach α = 0.90, with subscale reliabilities ranging from 0.887 to 0.894.)

All the above-mentioned English questionnaires were translated into Greek by two independent translators. The items in this reconciled translation were then back-translated to ensure consistency with the items of the English version. This comparison was designed to provide the final versions of the Greek FAS, Big Five, Loneliness, CDI (i.e. Depression) and Anxiety questionnaires. The entire procedure was monitored by two psychologists and one pedagogist, to ensure the reliability of the translated versions.

### Preliminary Data Analysis

The nature of our questionnaire, allows us to investigate and measure students’ behavioral insights related to the occurrence of Covid-19 through several ways. Our primary measurements are the level of students’ loneliness, current depression, current anxiety and general anxiety. Table 2 contains the summary statistics for all above variables, the t-tests of differences among males and females, direct comparisons of students from Junior High School and General High School and subjects with high and low average grades (Column 4).

**Table 2:** Outcomes Summary Statistics (Dependent Variables)

Gender	Pooled sample	Males	Females	Difference
	[1]	[2]	[3]	[4]: [3]-[2]
Loneliness Index	65.47	65.10	65.74	0.64
T_1_ Depression Index (CDI)	10.84	10.98	10.74	0.24
T_2_ Depression Index (CDI)	12.18	11.03	13.03	2.00^***^
Percentage Change of Depression (%)	26.63	7.29	40.75	33.46^***^
T_1_ Period Anxiety (T)	37.62	34.84	39.68	4.84^***^
T_2_ Period Anxiety (S)	33.04	31.84	33.93	2.09^***^
Percentage Change of Anxiety (%)	-9.31	-5.69	-11.98	-6.29^***^
Age	Pooled sample	Junior High school	General High school	Difference
	[1]	[2]	[3]	[4]: [3]-[2]
Loneliness Index	65.47	64.42	65.83	1.41^*^
T_1_ Depression Index (CDI)	10.84	11.03	10.77	-0.24
T_2_ Depression Index (CDI)	12.18	12.94	11.92	-1.02^*^
Percentage Change of Depression (%)	26.63	28.91	25.83	-3.08
T_1_ Period Anxiety (T)	37.62	36.23	38.11	1.88^***^
T_2_ Period Anxiety (S)	33.04	31.55	33.56	2.01^***^
Percentage Change of Anxiety (%)	-9.31	-10.14	-9.02	1.12
Grades	Pooled sample	Low Grades	High Grades	Difference
	[1]	[2]	[3]	[4]: [3]-[2]
Loneliness Index	65.47	63.31	66.05	2.74^**^
T_1_ Depression Index (CDI)	10.84	13.22	10.20	-3.02^***^
T_2_ Depression Index (CDI)	12.18	15.02	11.42	-3.60^***^
Percentage Change of Depression (%)	26.63	18.67	28.79	10.12
T_1_ Period Anxiety (T)	37.62	38.70	37.33	-1.37^*^
T_2_ Period Anxiety (S)	33.04	32.98	33.06	0.08
Percentage Change of Anxiety (%)	-9.31	-12.12	-8.55	3.57^**^

Author’s Calculations (Mean Scores)

The t-test is the statistics of contrast of a test of no-difference in means between students

^⁎⁎⁎^ Statistically significant at the 1% level

^**^ Statistically significant at the 5% level

^*^ Statistically significant at the 10% level

The Loneliness index has a min value of 21 and a max value of 80 and Std. Dev. 10.228

The T_1_ Depression (CDI) index has a min value of 0 and a max value of 32 and Std. Dev. 6.230

The T_2_ Depression (CDI) index has a min value of 0 and a max value of 43 and Std. Dev. 12.183

The Current Anxiety (S) index has a min value of 20 and a max value of 57 and Std. Dev. 6.526

The General Anxiety (T) index has a min value of 20 and a max value of 60 and Std. Dev. 8.983

The Percentage Change of Anxiety (%) has a min value of – 52.27% and a max value of 50% and Std. Dev. 18.895

The Percentage Change of Depression (%) has a min value of – 100% and a max value of 600% and Std. Dev. 84.795

Next, regarding the loneliness index, we observe an average value of 65.47 (the scale is from 20- low loneliness level to 80- high loneliness level), indicating a high prevalence of this unpleasant emotional response to home isolation and social distancing due to the Covid-19 pandemic. Concerning differences amongst individuals, Table 2 shows that there is no a statistical difference between the loneliness index in terms of gender, but students with high grades from General High Schools (aged 15–18 years) face a statistically significant greater level of this state of distress. In our analysis we utilize the logarithmic values of the loneliness index.

As regards students’ depression levels in the T2 Pandemic period, the results show an average of 12.18 and for period T1 and an average of 10.84 (the scale is from 0- low depression to 52- high depression), which are much higher than the previous research findings by Giannakopoulos et al., 2009, who, when conducting a large field experiment among Greek students, reported a mean score for current depression of 7.11. Moreover, similarly, to Giannakopoulos et al., 2009, we also find a statistically significant greater level on depression (CDI index) in female participants but only in the Covid-19 T2 period (13.03 vs 11.03 in males). In addition, our analysis also revealed that younger ages (Junior High School students) and students with low grades face greater levels of current depression (at 10% and 1% levels of statistical significance respectively). In our analysis we used the standardized values of the CDI index. We could not use logarithmic values because the initial variable also contained the zero value. To standardize the variable, we used the following formula z = (X-μ)/σ, where X is the original variable, μ is the mean and σ is the standard deviation. Regarding the percentage chance of the depression, we found an approximately 26.63% increase in depression, due to the pandemic-imposed life restrictions. This increase was significantly higher for female participants.

Concerning anxiety, we observe that its S-form (T2 Period) shows a mean value of 33.04 while its T-form (T1 period) a mean of 37.62 (scale from 0- low anxiety to 60- high anxiety). Similarly, to Psychountaki et al., 2003, who also conducted a large field experiment among Greek students, we found that general anxiety (T-Anxiety) is greater than current anxiety (S-Anxiety), with an average value of 37.62 (Psychountaki et al., 2003, reported a mean value of 35.21), with the T-Anxiety means of girls being significantly higher than the T-Anxiety means of boys (|t|= 5.65, *p* = 0.01). In this direction, the S-Anxiety means of girls is significantly higher than the S-Anxiety means of boys (|t|= 3.29, *p* = 0.01). Table 2 also revealed that older students face greater levels of both forms of anxiety at 1% level of significance. In our analysis, we made use of the logarithmic values of both T-Anxiety and S-Anxiety.

Deepening our analysis, as our study revealed higher levels of anxiety in the pre-Covid-19 period compared to Psychountaki’s report on the Greek student population (i.e. 33.04 vs 27.98), to investigate the behavioral insight of students we initially constructed the percentage change of Anxiety (%). In general, in our sample only 31.7% of students (133 out of 419), displayed an increase of anxiety due to Covid-19 and everyday life restrictions. Going back to Table 2, we find an average decrease of anxiety by 9.31% and a statistically significant difference between the percentage change of anxiety in terms of gender, with females displaying approximately 12% lower anxiety and males 5.7% respectively. In relation to cognitive differences, we observe that students with poor grades display a 12.12% decrease, while those with good grades a 8.55% decrease (|t|= 1.58, *p* = 0.05), indicating possible sample variation on levels of emotional self-efficacy (Galla & Wood, 2012).

In our regressions, we embed the standardized relative forms of anxiety and depression change, because the percentage change assumes both an absolute zero (which does not exist in these types of ratings), as well as equal intervals (namely that difference between 1 and 2 is the same as between 3 and 4), which is rarely true for psychological variables.

**Fig. 2:**
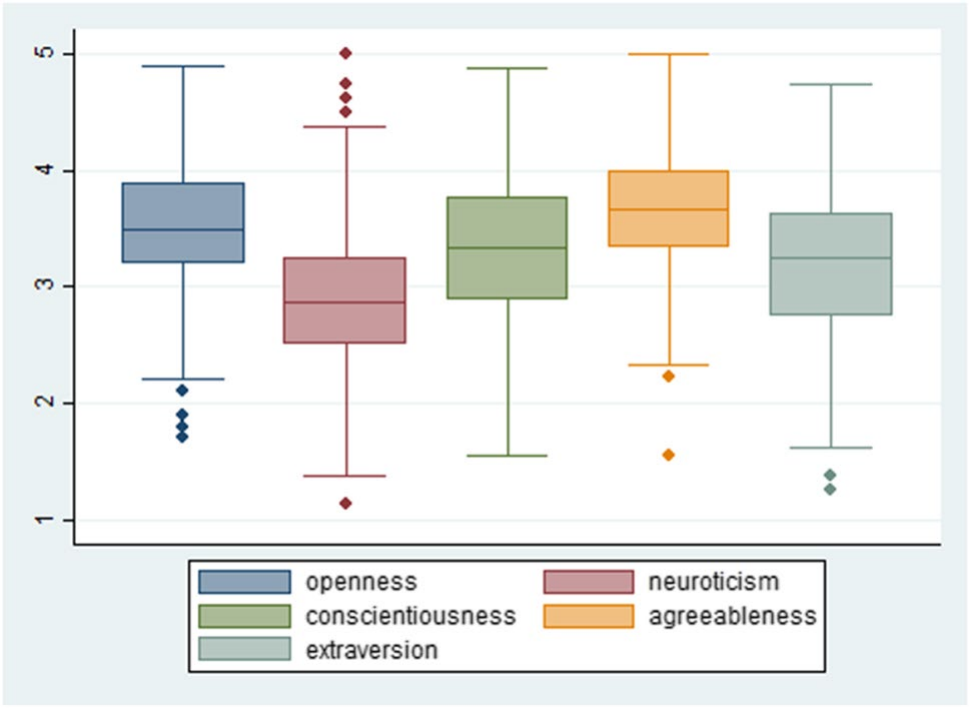
Personality traits Distribution plots with outliers

According to the observed variation in personality traits, we notice that the mean score for Openness is 3.552, suggesting that our sample consists of students with a high tendency toward creativity and active imagination, 3.351 for Conscientiousness signifying high levels of thoroughness, 3.173 for Extraversion exhibiting a satisfactory level of energetic student behavior, 3.640 for Agreeableness indicating that our participants seem to be more empathetic and altruistic and 2.896 for Neuroticism suggesting that our sample does not tend to experience negative emotions in general. We did not notice a great number of extreme values for any of the above personality variables (Fig. 2).

**Fig. 3:**
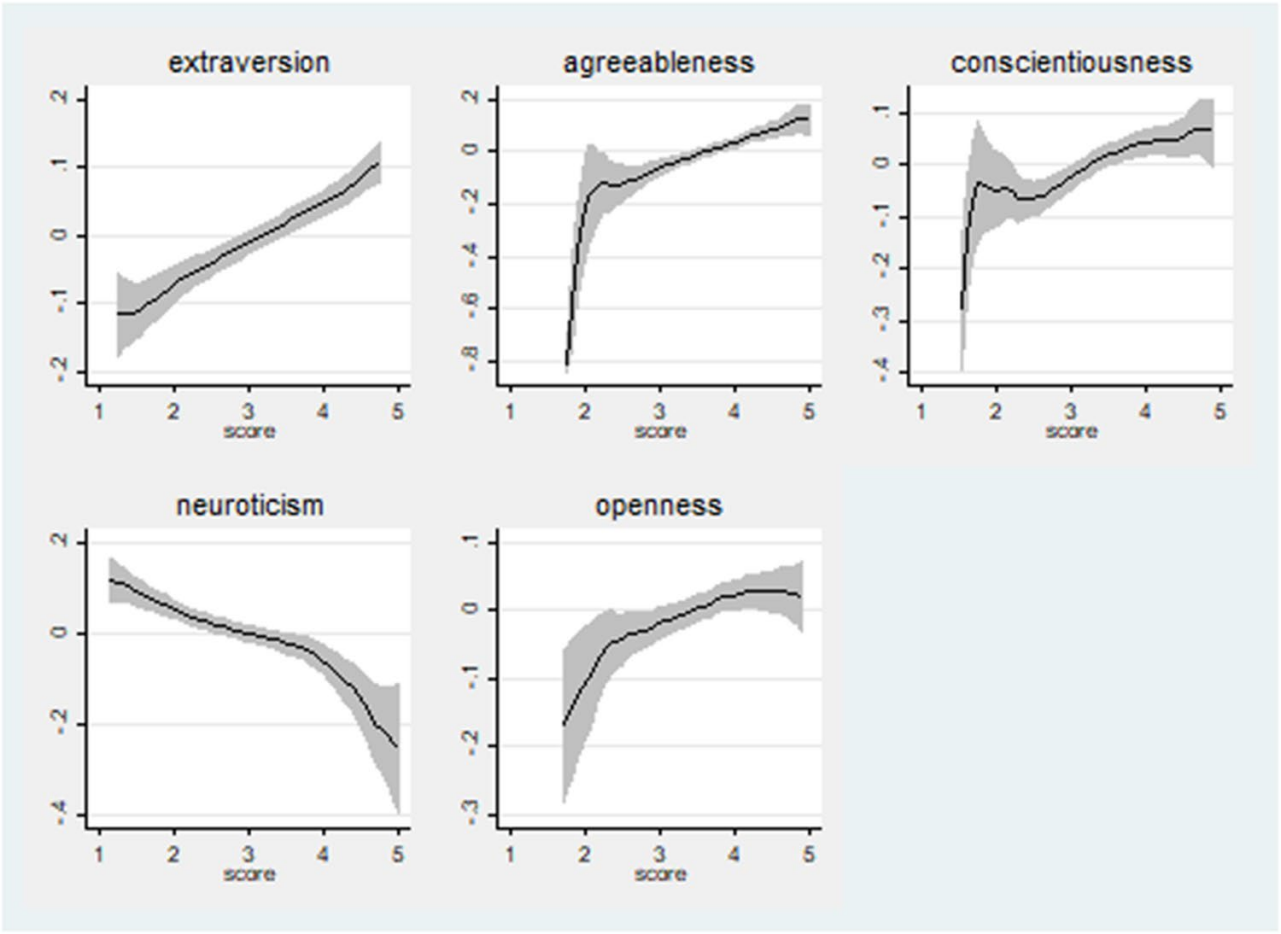
The relationship between personality traits and students’ loneliness level. Source: Dataset with results drawn from the Questionnaire (N = 419). Author’s calculations. Notes: Local polynomial smoothing with confidence bands (shaded area)

**Fig. 4:**
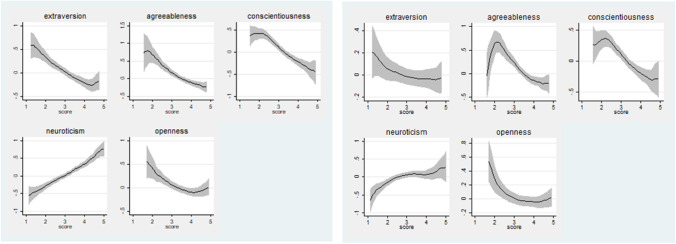
The relationship between personality traits and students’ depression levels on T2 –Pandemic and T1 pre Pandemic periods. Source: Dataset with results drawn from the Questionnaire (*N* = 419). Author’s calculations. Notes: Local polynomial smoothing with confidence bands (shaded area)

**Fig. 5:**
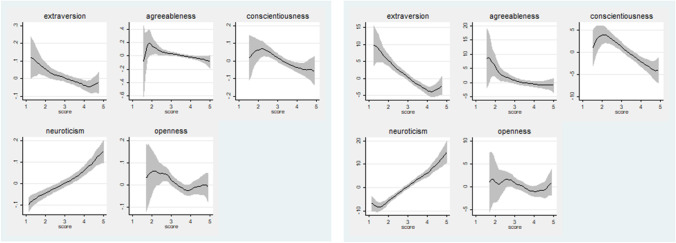
The relationship between personality traits and students’ anxiety (S) and anxiety (T) levels (T2 and T1 periods respectively). Source: Dataset with results drawn from the Questionnaire (*N* = 419). Author’s calculations. Notes: Local polynomial smoothing with confidence bands (shaded area)

Additionally, to understand the link between a student’s loneliness, depression and anxiety with personality traits, in Figs. 3, 4 and 5 we present a vivid representation the relationships between our sample’s levels of loneliness, depression and anxiety and the distribution of each personality trait, using non-parametric local polynomial smoothing techniques. We observe that the degree of a student’s sense of loneliness is positively related with extraversion, agreeableness, conscientiousness and openness and negatively only with neuroticism (Fig. 3).

In terms of depression in the Covid-19 period, we also notice that the degree of a student’s CDI index, in both cases, is positively related only with neuroticism and negatively with levels of extraversion, agreeableness, conscientiousness and openness (Fig. 4).

Finally, Fig. 5 illustrates that for both anxiety formations, only the trait of neuroticism has a positive linear relationship.

## Empirical Model

Thus, a simplified generic cognitive vulnerability-stress model was utilized, stating that a negative event contributes to several psychological disorders and symptoms (especially when moderated by cognitive vulnerability), such as anxiety, depression and loneliness. For estimation purposes, we rely on typical regression models where specific disorder indicators are utilized as the dependent variable. To isolate the effects of personality traits and other “environmental” factors, the following econometric specification was utilized:

1 $${PDi}={\alpha }+{\beta Di}+{\gamma Pik}+{\delta Ci}+{\zeta Fi}+{ei}$$

where PDi is a specific psychological disorder (i.e. loneliness, depression, anxiety) indicator for student i, Di is a vector of the demographic (i.e. gender, age) characteristics of student i, Pik is a k-vector of non-cognitive personality traits of student i (where k = 1,…,5 corresponds to Openness, Conscientiousness, Extraversion, Agreeableness and Neuroticism, respectively), Ci is a vector that includes cognitive indicators for student i regarding school performance and computer competence, Fi is a vector of the family and social economic background characteristics of student i (including the satisfaction with life index, family affluence index, parental status, number of brothers and sisters, area of residence), and ei the disturbance error term, where E[ ei | xi] = 0 and Var[ ei | xi] = σ2.

Although general, this specification, described by Eq. (1), is expected to provide evidence of the role of Big Five variables (standardized to have a zero mean and a standard deviation of one) on students’ levels of loneliness, depression and anxiety through the vector of the estimated coefficients γk (using linear regression techniques). The dependent variable PDi refers to (a) the logarithmic values of the loneliness index (b) the standardized values of the depression (CDI) index in two time periods, (c) the logarithmic values of the anxiety (S) and anxiety (T) (two time periods), (d) the standardized values of the anxiety change (PCA index) and the standardized values of depression change (PDC index).

## Results

### Baseline

Table 3 presents our baseline estimates on the impact of personality traits on the log of loneliness (column 1), on the standardized values of depression in the period before the Pandemic (column 2), on the standardized values of depression during the Pandemic (column 3) and the log values of the aspects of anxiety before and during this (column 4 and 5 respectively). Many of our findings agree with the results obtained in both economics and psychology literatures.

**Table 3:** Determinants of Outcome Variables

	Logarithmic Values of	Standardized Values of	Standardized Values of	Logarithmic Values of	Logarithmic Values of
	Loneliness (T_2_ Period)	Depression (T_1_ Period)	Depression (T_2_ Period)	General Anxiety (T_1_ Period)	Current Anxiety (T_2_ Period)
	[1]	[2]	[3]	[4]	[5]
Constant	3.984^***^ (0.050)	1.537^***^ (0.310)	1.483^***^ (0.242)	3.729^***^ (0.058)	3.651^***^ (0.056)
Demographics					
Female	0.022 (0.015)	0.085^*^ (0.084)	0.118^*^ (0.068)	0.079^***^ (0.017)	0.029^*^ (0.017)
General High School	0.013 (0.017)	0.167 (0.105)	0.082 (0.084)	0.083^***^ (0.021)	0.080^***^ (0.018)
Big Five Personality Traits					
Openness	-0.014 (0.008)	0.024 (0.051)	0.069 (0.043)	0.018^*^ (0.010)	-0.004 (0.008)
Conscientiousness	0.003 (0.008)	-0.139^***^ (0.049)	-0.137^***^ (0.036)	-0.018* (0.010)	0.007 (0.010)
Extraversion	0.050^***^ (0.007)	0.028 (0.046)	-0.137^***^ (0.038)	-0.041^***^ (0.009)	0.004 (0.009)
Agreeableness	0.053^***^ (0.008)	-0.192^***^ (0.046)	-0.164^***^ (0.034)	0.019^*^ (0.011)	-0.007 (0.009)
Neuroticism	0.020^**^ (0.008)	0.063^*^ (0.050)	0.289^***^ (0.039)	0.107^***^ (0.009)	0.059^***^ (0.009)
Cognitive Skills					
High Grades	0.012 (0.017)	-0.263^**^ (0.104)	-0.305^***^ (0.088)	-0.047^**^ (0.022)	0.001 (0.018)
Computers & Smartphone Competence	0.004 (0.003)	-0.024 (0.018)	-0.019 (0.014)	-0.003 (0.003)	0.002 (0.003)
Social Economic Background					
Satisfaction with life index (SWLS)	0.006^***^ (0.001)	-0.057^***^ (0.009)	-0.058^***^ (0.007)	-0.010^***^ (0.002)	-0.011^***^ (0.002)
High Family Affluence index (FAS)	0.016 (0.015)	-0.043 (0.089)	-0.035 (0.071)	-0.021 (0.018)	-0.017 (0.016)
Urban Family Area	-0.023 (0.014)	0.019 (0.081)	-0.058 (0.064)	-0.010 (0.017)	-0.001 (0.015)
*Parental Status*					
Single Mother	0.025 (0.023)	0.312^**^ (0.141)	0.246^**^ (0.113)	0.002 (0.029)	-0.008 (0.028)
Single Father	0.044 (0.032)	-0.222 (0.234)	-0.149 (0.171)	0.011 (0.048)	-0.150^***^ (0.034)
Other Relative	0.039^*^ (0.024)	-0.597^**^ (0.267)	-0.209 (0.195)	-0.010 (0.060)	-0.034 (0.040)
*Number of Brothers and Sisters*					
1	0.004 (0.023)	-0.117 (0.151)	-0.043 (0.118)	0.006 (0.034)	-0.005 (0.027)
2	0.002 (0.026)	-0.152 (0.161)	-0.070 (0.129)	0.017 (0.037)	-0.047 (0.031)
3	-0.033 (0.039)	0.196 (0.196)	0.065 (0.157)	0.085^*^ (0.044)	0.035 (0.037)
4	-0.058 (0.047)	0.061 (0.323)	0.016 (0.266)	0.147^**^ (0.065)	0.036 (0.074)
5 and more	-0.153 (0.141)	0.124 (0.565)	0.816^**^ (0.356)	0.183^*^ (0.107)	-0.008 (0.131)
R^2^	0.388	0.359	0.592	0.524	0.361
F-Stat	10.90	9.22	26.24	27.82	12.84

Authors’ Calculations. Data drawn from the Online Questionnaire

The number of the participant subjects is N = 419. High FAS index is a median split dummy from the questionnaire survey measure. For parental status the reference group is the Both Parents category. For the number of brothers’ and sisters’ variable the reference group is the single child status (i.e. 0 number of brothers and sisters). For General High School the reference group is Junior High School

Robust standard errors in parentheses. Statistical Significance: *** *p* < 0.01, ** *p* < 0.05, *p < 0.10

Regarding the loneliness index, a student’s sense of loneliness has a positive, statistically significant relationship with the traits of extraversion, agreeableness and neuroticism. An increase of a standard deviation in the level of extraversion is correlated with a 5.0% increase in loneliness. Similarly, an increase of a standard deviation in the level of agreeableness and neuroticism has a positive impact of 5.3% and 2.0% in students’ loneliness, at 1% level of significance. Interestingly, a student’s level of satisfaction with life in general has a strong significant effect on loneliness and only students living with other relatives have a significant higher sense of loneliness in relation to students in nuclear families (Column 1, Table 3). It seems that mainly extraversion and agreeableness function protectively on participants’ sense of loneliness due to the pandemic.

In respect to adolescents’ depressive symptoms, as expected, depression rates are higher in females than in males, confirming previous studies on gender differences and the prevalence of depressive symptoms (Bebbington, 1996; Nolen-Hoeksema, 1987) and our H1 hypothesis. Regarding the students’ personality profile, the coefficients of the big five traits revealed a positive relationship between depression and neuroticism and a negative relationship with conscientiousness and agreeableness at 1% level of significance. Thus, agreeable or conscientious students seem to deal better with the life changes due to Covid-19 (such as home isolation and social distancing) compared to neurotic students. These effects hold also in the second wave of our research, with the effect and magnitude of neuroticism being more robust and increased in the T2 period (from 0.063 to 0.289). Moreover, the effect of extraversion on depression seems to emerge only in the T2 period, confirming the H3 hypothesis, only as time within the pandemic period progresses.

Lastly, it is notable that students with high grades and students with high levels of satisfaction with life scored less in the current depression scale, while depression was significantly higher in students from a single-mother family than those from nuclear families (columns 2 and 3).

Table 3 also reports the two measures of anxiety available in our online questionnaire: metrics for participants’ current anxiety (S) levels during the Pandemic in column (5) and their general anxiety (T) levels (i.e. initial anxiety level) in column (4). While in the general anxiety specification personality facets seems to play a significant role, this does not apply in the current anxiety specification, in which only neuroticism has a positive significant correlation. More particularly, a one standard deviation increase in a student’s level of neuroticism is associated with an increase in current depression of about 5.9%. Females scored approximately 3% higher on current anxiety than males, while students from single-father families scored 15% less than those from nuclear families (H5 hypothesis).

Furthermore, in terms of general anxiety (column 4), several estimations of our specification are in line with previous research. This study also finds that girls report approximately 8% higher anxiety than boys (Rodrigues et al., 2018). As regards personality, the findings also confirmed the robust positive effect of neuroticism on general anxiety and the negative effect of extraversion (Saklofke et al., 1995; Costa and McCrae, 1992; Rusting & Larsen, 1998). More specifically, an increase of one standard deviation in the level of neuroticism is correlated with a 10.7% increase in general anxiety, while an increase of one standard deviation in extraversion is associated with a 4.1% decrease in general anxiety, at 1% level of significance. Our analysis also reveals evidence on the effect of conscientiousness on health behaviors such as anxiety (Hampson et al., 2007, 2010; Mendolia & Walker, 2014a, b), as a one standard deviation rise in a student’s level of conscientiousness is associated with a decrease in general anxiety of about 2.0%. As a final point, a weak positive effect of openness and agreeableness on general anxiety exists, increasing its value by approximately 2%. For both forms of anxiety, older students (from General High Schools) reported approximately 8.0% higher anxiety levels. A possible interpretation is that this is due to the Greek educational system, in which National examinations after General High School determine admission to higher educational forms (university etc.). Lastly, for both anxiety specifications, a student’s level of satisfaction with life had a negative impact with a similar magnitude (approximately 1.0%).

### Changes in Anxiety and Depression

The core issue investigated in this research is the mechanisms underlying changes in depression and anxiety due to the pandemic. The survey was thus conducted in two waves. Having the relative change in participants’ depression and anxiety levels standardized and embedded in our analysis revealed some interesting findings regarding the effects of personality traits. Table 4 includes the regressions for the prediction of the relative change in anxiety (column 1) and depression (column 2).

**Table 4:** Determinants of the percentage change of Anxiety and Depression levels

	Standardized Change of Anxiety [1]	Standardized Change of Depression [2]
Constant	-0.468^**^ (0.240)	0.412^***^ (0.151)
Demographics		
Female	-0.222^***^ (0.077)	0.224^***^ (0.046)
General High School	-0.035 (0.081)	-0.071 (0.057)
Big Five Personality Traits		
Openness	-0.091^**^ (0.042)	0.056^**^ (0.029)
Conscientiousness	0.088^*^ (0.048)	-0.021 (0.028)
Extraversion	0.190^***^ (0.041)	-0.187^***^ (0.026)
Agreeableness	-0.094^**^ (0.047)	0.001 (0.031)
Neuroticism	-0.213^***^ (0.043)	0.275^***^ (0.031)
Cognitive Skills		
High Grades	0.204^**^ (0.088)	-0.093 (0.064)
Computers & Smartphone Competence	0.024 (0.016)	0.003 (0.009)
Obs	419	419
R^2^	0.246	0.470
F-Stat	9.22	9.59

Authors’ Calculations. Data drawn from the Online Questionnaire

All specifications control for students’ social economic attributes and family characteristics. For General High School the reference group is Junior High School

Robust standard errors in parentheses. Statistical Significance: *** *p* < 0.01, ** *p* < 0.05, *p < 0.10

Surprisingly, our results also reveal a differential effect of gender on anxiety and depression. Although females initially reported higher anxiety and depression levels (Table 3) during the pandemic, they seem to adapt better to life changes (home isolation and social distancing) than males, in terms of anxiety (22.2% lower than males) (Table 4, column 1), while their depression levels remain higher (22.4% higher than males). At this point our study confirms Hypothesis H1, with the gender being a moderator of the effects of personality traits on anxiety and depression.

Moreover, evidence suggests that, mainly the traits of extraversion and neuroticism predict the relative change in adolescents’ mental health issues. Extraversion exerts a robust positive effect on anxiety and a negative one on depression, while neuroticism follows the opposite course. An increase of one standard deviation in the level of extraversion is correlated with a 19% increase in the anxiety change index and an 18.7% decrease in depression, while an increase of one standard deviation in neuroticism is correlated with a 21.3% decrease in the anxiety change index and a 27.5% increase in depression, at 1% level of significance (H2, H3 hypotheses). Our specifications also reveal the effects of openness on both relative change of anxiety and depression and agreeableness and conscientiousness only on the participants’ relative change of anxiety, effects with a small magnitude and weak statistical significance (H4 Hypothesis).

Finally, the results also confirm the effect of the cognitive index (i.e. high grades) only for the case of anxiety. It appears that students with high grades face higher levels of anxiety during the pandemic (H5 hypothesis).

### Heterogeneity

**Fig. 6:**
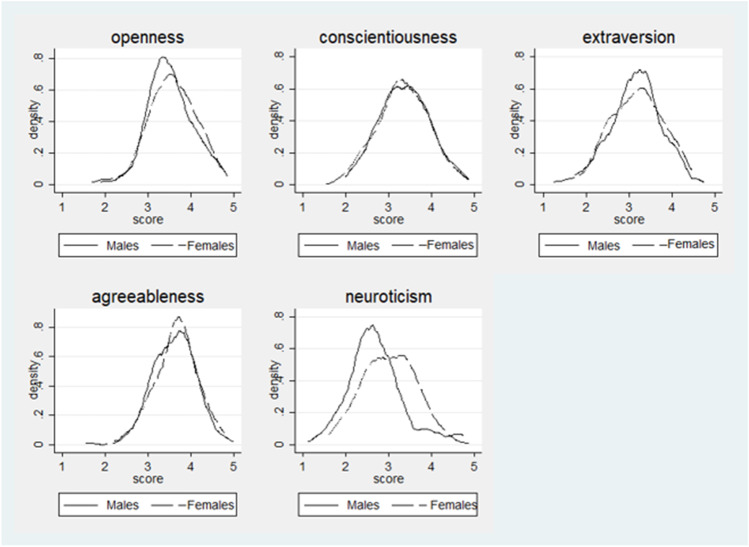
Personality Traits Density Distribution by gender

**Fig. 7:**
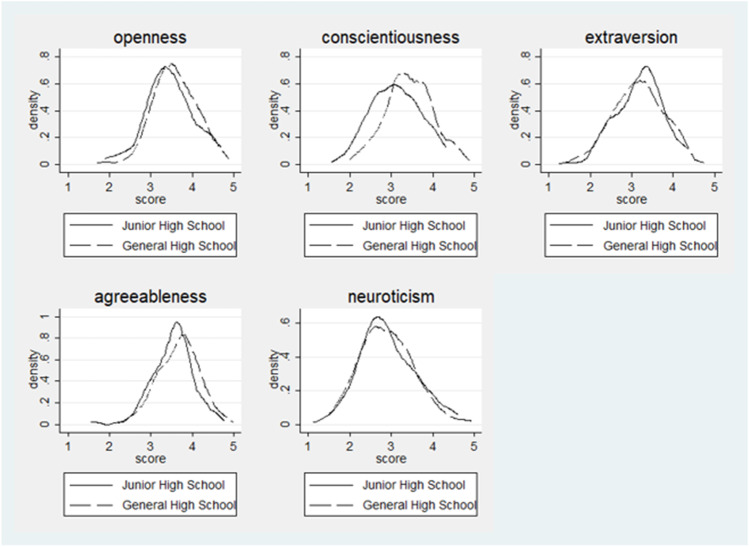
Personality Traits Density Distribution by age

We now turn our attention to the possibility that personality may be associated with anxiety and depression differently in various subsamples. This paper examines whether students of different genders and age groups present different effects of personality traits on health behavior, resulting in anxiety and depression during the Covid-19 pandemic. In addition, there are some significant differences in the distribution of traits by major. Initially, evidence exists that females and males differ in health behavior (Table 2), but there are also significant gender differences in the distribution of soft skills (i.e. personality traits). Statistical two sample t-tests suggest that girls are more neurotic than boys (|t|= 4.811 & *p* value = 0.000) (see also Fig. 6 in Appendix 1). Furthermore, concerning age, older students, from General High School, have a higher tendency to openness to new experiences (|t|= 1.876 & *p* value = 0.030), are more conscientious ((|t|= 5.092 & *p* value = 0.000) and also tend to be more agreeable (|t|= 2.606 & *p* value = 0.004) than Junior High School students (see also Fig. 7 in Appendix 1). These results are mostly consistent with several studies on gender differences in personality traits (Costa et al., 2001; Schmitt et al., 2008; Cubel et al., 2016). In the following tables, this paper thus also explores whether the relationship between students’ personality and our basic outcome variables (i.e. standardized relative change of anxiety and depression) differs due to gender and age.

Hence, Table 5 presents our first set of heterogeneous effects for students’ standardized relative changes in anxiety. Column 1 includes the baseline results for ease of comparison in each case. Column 2 allows the impact of personality traits to vary between males and females, column 3 presents the results when effects are allowed to vary by age and column 4 is the full specification, which includes all interactions.

**Table 5:** Heterogeneity by Gender, Age and Grades

Y: Standardized Change of Anxiety	(1)	(2)	(3)	(4)
Openness	-0.091^**^ (0.042)	-0.158^***^ (0.061)	-0.42 (0.066)	-0.107 (0.089)
Openness x female		0.102 (0.086)		0.088 (0.087)
Openness x General High School			-0.065 (0.084)	-0.059 (0.085)
Conscientious	0.088^*^ (0.048)	0.134^**^ (0.062)	0.015 (0.081)	0.065 (0.096)
Conscientious x female		-0.070 (0.087)		-0.062 (0.088)
Conscientious x General High School			0.098 (0.099)	0.086 (0.099)
Extravert	0.190^***^ (0.041)	0.245^***^ (0.051)	0.201^**^ (0.095)	0.286^***^ (0.114)
Extravert x female		-0.092^*^ (0.076)		-0.103^*^ (0.078)
Extravert x General High School			-0.019 (0.104)	-0.049 (0.110)
Agreeables	-0.094^**^ (0.047)	-0.148^**^ (0.060)	-0.091 (0.092)	-0.161^*^ (0.104)
Agreeables x female		0.085 (0.090)		0.097 (0.091)
Agreeables x General High School			0.001 (0.107)	0.013 (0.107)
Neurotics	-0.213^***^ (0.043)	-0.213^***^ (0.060)	-0.243^***^ (0.079)	-0.232^***^ (0.093)
Neurotics x female		-0.009 (0.082)		-0.010 (0.081)
Neurotics x General High School			0.037 (0.088)	0.023 (0.089)
N	419			
F-Stat	9.22	7.88	7.31	6.31
R^2^	0.246	0.259	0.249	0.256

Authors’ Calculations. Data drawn from the Online Questionnaire

Notes Outcome variable is the standardized value of the change in Anxiety due to the Pandemic. All specifications control for students’ demographics, cognitive abilities, social economic attributes and family characteristics. For General High School the reference group is Junior High School

Robust standard errors in parentheses. Statistical Significance: *** *p *< 0.01, ** *p* < 0.05, *p < 0.10

Interestingly, the results show that openness, conscientiousness, extraversion and neuroticism have differential effects on the relative change in anxiety only for boys (column 2), and that extraversion and neuroticism have a statistically significant impact on anxiety for the lower ages in the sample (column 3). The effects that hold in the full heterogeneity specification state that a rise of one standard deviation in extraversion increases the relative change of males’ anxiety by 28.6% at 1% level of significance (point estimate for males 0.286) while the net effect for females is approximately -10% (point estimate for females -0.103). This suggests that the detrimental effect of extraversion on anxiety is mainly driven by gender composition (column 4). Furthermore, following Table 5, neuroticism is negatively correlated with males, an effect that remains robust in all specifications. A rise of one standard deviation in the level of neuroticism causes an anxiety recession only for males, by approximately 23% (point estimate for males -0.232) (column 4).

Following the same strategy for heterogeneity effects, Table 6 presents the same set of heterogeneous effects for students’ relative change in depression. Concerning the gender composition of effects on depression, column 2 shows that the trait of neuroticism correlates positively for both genders, extraversion correlates negatively only for males and openness correlates with depression differently (i.e. positive correlation for males and negative correlation for females). Regarding the indicator of age, column 3 reveals that extravert junior students have lower levels of relative changes in depression due to the pandemic that older students. In addition, junior students self-reported as neurotics have an approximately 44% increase in depression, while older ones face a decrease of approximately 23% (point estimate for older students -0.226) at 1% level of significance. Lastly, to draw some conclusions for the sources of the effects, column 4 includes all interaction terms. Thus, it seems that extraversion has a standalone effect on depression only for males, regardless the age composition of the sample. A rise of one standard deviation in the extraversion score for boys is correlated with a 12.3% increase in depression. Furthermore, the effect of openness is gender-dependent, as an increase of one standard deviation in its score increases depression by approximately 15% for males and decreases depression by approximately 12% for females. Finally, the effect of neuroticism is different across genders and ages. An increase of one standard deviation in neuroticism for a junior male is correlated with a 26.1% increase in depression changes, while this effect is negligible for older females and negative by almost 16% (point estimate -0.157). This suggests that the detrimental effect of neuroticism on depression changes is not only driven by the gender composition of age. No relation between changes in depression and the other personality traits exist (column 4).

**Table 6:** Heterogeneity by Gender, Age and Grades

Y: Standardized Change of Depression	(1)	(2)	(3)	(4)
Openness	0.056^**^ (0.029)	0.142^***^ (0.038)	0.049 (0.050)	0.147^***^ (0.057)
Openness x female		-0.116^**^ (0.055)		-0.122^**^ (0.055)
Openness x General High School			-0.002 (0.060)	-0.012 (0.059)
Conscientious	-0.021 (0.028)	-0.039 (0.033)	-0.027 (0.058)	-0.055 (0.059)
Conscientious x female		0.015 (0.047)		0.025 (0.047)
Conscientious x General High School			0.012 (0.066)	0.016 (0.062)
Extravert	-0.187^***^ (0.026)	-0.170^***^ (0.040)	-0.153^***^ (0.061)	0.123^**^ (0.066)
Extravert x female		-0.018 (0.054)		-0.038 (0.052)
Extravert x General High School			-0.053 (0.068)	-0.051 (0.064)
Agreeables	0.001 (0.031)	0.006 (0.034)	-0.056 (0.081)	-0.061 (0.074)
Agreeables x female		-0.030 (0.058)		0.123 (0.055)
Agreeables x General High School			0.071 (0.086)	0.077 (0.081)
Neurotics	0.275^***^ (0.031)	0.125^**^ (0.034)	0.439^***^ (0.068)	0.261^***^ (0.065)
Neurotics x female		0.273^***^ (0.059)		0.240^***^ (0.056)
Neurotics x General High School			-0.213^***^ (0.076)	-0.157^**^ (0.069)
N	419			
F-Stat	9.59	10.35	8.75	9.77
R^2^	0.470	0.519	0.492	0.533

Authors’ Calculations. Data drawn from the Online Questionnaire

Outcome variable is the standardized value of the change in Depression due to the Pandemic. All specifications control for students’ demographics, cognitive abilities, social economic attributes and family characteristics. For General High School the reference group is Junior High School

Robust standard errors in parentheses. Statistical Significance: *** *p* < 0.01, ** *p* < 0.05, *p < 0.10

### Discussion

This article deals with the effects of personality traits on adolescents’ levels of loneliness, anxiety and depression. Our target group was in the 12–18 age group. Thus, this study randomly recruited 419 Greek secondary school students. The research was conducted in the beginning and in the middle of the Covid-19 pandemic in Greece, and after the public policies intended to induce a behavioral change with measures against interpersonal contacts (i.e. home isolation and social distancing).

The key predictors of the level of perceived threat by the Coronavirus were extraversion and neuroticism. Originally proposed as one of the key personality dimensions by Eysenck (1991), extraversion and neuroticism also form part of the Big-5 personality theory, the reliability and validity of which has been well documented in countless cultures around the globe (Costa & McCrae, 1992).

The results indicate that extraversion predicts the 19% increase in the relative change in students’ anxiety, and the 18.7% decrease in students’ relative change in depression. On the other hand, neuroticism seems to explain the 21.3% of dropping anxiety levels and the 27.5% increase in depression.

In general, although the increase in depression (by 26.63%) was expected, surprisingly, our results revealed that, on average, the students’ anxiety levels decreased by 9.31%. The study was conducted in two waves (panel data). This allows us to measure mental health outcomes not only based on determinants during the investigation period but also based on personality determinants of relative changes caused by life restrictions due to the Pandemic. Hence, our findings do not suffer from cross-sectional investigation limitation (Pierce et al., 2020a, b). These results were a "big surprise" and raised questions about the impact of the school environment on teenagers' mental health.

The findings also showcase several concerns about the way of life of Greek adolescents. The small number of confirmed cases and deaths from Covid-19 in Greece and the early adoption of restrictive measures by the Greek authorities undoubtedly play an important role in preventing students from perceiving Covid-19 as a highly infectious, fatal disease. Another interpretation is that, before the Covid-19 pandemic, students were had high levels of anxiety due to the structure of the educational system in Greece and the continuous and increased pressure it exerted on them. It is well known that the Greek educational system offers substantial educational mobility, with free education for all, but, due to inequalities and qualitative differences in the secondary public schooling system, nowadays private tutoring plays an important role (Daouli et al., 2010; Tsakloglou & Antoninis, 1999). Hence, during the pandemic, students may find the opportunity to calm down. However, it is obvious that their physical isolation has a varied impact on their behavior, resulting in loneliness, anxiety and depression, depending on their personality traits. Personality is understood from a variety of theoretical perspectives as a useful conceptual framework with five measurements (i.e. Big Five Personality traits). Each of these has a unique contribution to our understanding of individual differences in behavior and experience.

Thus, as expected, these results also showed that mainly students with high levels of neuroticism, extraversion and agreeableness face a sense of loneliness, which may evolve into anxiety or depression over time (we used loneliness as a proxy index of anxiety and depression, to investigate potential students with as yet unmanifested anxiety and depression symptoms)(Cuesta & Budría, 2015 and Lima et al., 2020).

Despite the fact that our sample is limited to secondary school students (Simons et al., 2017), we argue that our research provides valuable insights and contributes to the literature by highlighting the relevance of heterogeneity in individual characteristics and personality in the design of policy interventions aimed to confront a negative life event. For behavioral-based epidemiological interventions for infectious diseases, it is important to consider how policies interact with individual personality characteristics. While it is not always the case that non-targeted policies will make society worse off than decentralized decision-making, it is clear that targeted policies would lead to greater benefits, and part of the reason that targeting is so important is to better understand the role of non-cognitive traits on mental health behaviors and the personality profile of those mostly -positively or negatively- affected by major and wide-spread negative events such as a pandemic.

Our research allows further investigation for a deeper understanding of personality from a neurobiological perspective, focusing on the biochemistry of behavioral systems in reactions to life conditions due to major negative events and trying to give insights to the question: How much of our personality’s reaction to such negative events is due to innate biological aspects, and how much is influenced by the environment and culture we are raised in? (Laliotis & Minos, 2020; Steel et al., 2008).

The study limitations include the fact that our sample is not representative at national level, although we did have participants from all regions of Greece. Secondly, although the survey was conducted in two different periods (panel data), it cannot be considered a longitudinal investigation. Thus, our findings cannot be efficiently compared to longitudinal mental health studies during the COVID-19 Pandemic (Pierce et al., 2020a, b; Twenge & Joiner, 2020), but add useful insights on the ongoing research of the pandemic’s consequences for life (Parlapani et al., 2020; Peppou et al., 2021; Siettos et al., 2021). Finally, due to the survey’s open call procedure, our results may suffer from self-selection bias and sample identification, because, although the survey followed a randomized distribution, the exact cognitive and non-cognitive characteristics and motivation of respondents and the profile of those who rejected our research call remains unclear (Bethlehem, 2010; Eysenbach & Wyatt, 2002).

## Conclusions

This paper provides evidence on the importance of considering personality traits as a relevant predictor of individual differences in health conditions during confinement due to the pandemic. It also aimed to provide insights on how personality in relation to demographic factors (i.e. gender, age), dynamically influence the mental health behavioral response caused by the pandemic. Neuroticism and extraversion outweighed the contribution of other important indicators. These findings have important theoretical and practical implications. On the one hand, they could help better understand mental health issues caused by the pandemic, and, on the other, considering personality traits is clinically useful for diagnosis and for planning personalized treatments and predicting their results more efficiently. The data also raise practical points, which governments should consider in order to decrease the public’s fear of COVID-19, including a push for clear public messages concerning the virus, stronger quality assurance mechanisms among media outlets, to promote objectivity and reduce the prevalence of “fake news”, and increased promotion of – and support for – mental health organizations, which have a valuable role to play in helping the public to manage anxiety and depression during this period (Brailovskaia et al., 2021; Niziurski & Schaper, 2021) e.g. (Fig. 6 and 7).

## Data Availability

Research data are shared upon request.

## Appendix 1

This section illustrates several heterogeneity graphs between the Big Five personality traits and gender (i.e. males, females) and age (i.e. students in Junior High schools and General High School).

## Footnotes

1 The evidence on the effect of personality on health has suggested, in general, that the personality traits of conscientiousness, openness to experience, and agreeableness generally have a positive effect on longevity and health behaviors (Hampson et al., 2007; Hampson et al., 2010; Mendolia & Walker, 2014a, 2014b). However, when it comes to extraversion and neuroticism, findings are controversial. Previous research has shown a strong positive correlation between depressive symptoms and neuroticism (Larsen, 1992) while other research has reported on the negative correlation between depression, anxiety and extraversion (Saklofke et al. 1995). A negative correlation between extraversion and depression has not always been found; for example, Costa and McCrae (1989) only found a positive correlation between extraversion and a positive mood. Other authors have obtained similar results: Extraverts show more emotional reactivity to a positive but not to a negative mood, whereas introverts show heightened emotional reactivity to a negative but not to a positive mood (Larsen & Ketelaar, 1989 and Rodrigues et al., 2018). Consistent with previous research, del Barrio et al., 1997, revealed that when the effects of gender and educational attainment on depression and anxiety are considered jointly, it was verified that girls with higher schooling performance obtain higher neuroticism scores, and also had higher depression and anxiety levels. On the other hand, a significant negative relationship among extraversion and depression was also revealed. Further investigations of extraversion and neuroticism in relationship to happiness and depression found that people who scored high in satisfaction appeared to be extraverted and internally controlled, and this also emphasized the dominant role of neuroticism over extraversion in explaining depression and anxiety (Rogalski & Paisey, 1987; Ormel & Wohlfarth, 1991; Rusting & Larsen 1998 and Pawar, 2018).

2 In Greece, secondary school education includes two sub-stages: compulsory “Junior High school”, also known as Gymnasium, which covers three years, for students aged 12 to 15 years and non-compulsory “General High school”, also known as General Lyceum, which also covers three years, for students aged 15 to 18 years. Although upper-level secondary education is not compulsory in Greece, most students attend the General High School, with a view to pursuing Higher Education studies. Nevertheless, the structure of the Greek educational system and its low quality state-sponsored education lead most students to resort to different forms of out‐of‐school support (Sianou-Kyrgiou, 2008). In literature, this private supplementary tutoring is usually termed *shadow education* (Lee et al., 2012). An important reason for students’ extensive resort to this is their orientation to several emerging online educational systems (Kassotakis & Verdis, 2013). These technologies have brought additional forms of tutoring through computer-assisted learning that may or may not include live contact with a tutor (Bray & Kobakhidze, 2014).
